# Meloside A Protects Dermal Papilla Cells from DHT-Induced Damage via Androgen Receptor Modulation

**DOI:** 10.3390/cimb47060436

**Published:** 2025-06-09

**Authors:** Hyun Jun Park, Bong Geun Song, Ji Hoon Song, Seung Hee Park, Da Hye Wang, Ho Kyun Kwon, Ji-Ean Lee

**Affiliations:** 1 R&D Center, Morechem. Co., Ltd., Yongin 16954, Gyeonggi-do, Republic of Korea; hjpark@morechem.net (H.J.P.); bgsong@morechem.net (B.G.S.); hgsong@morechem.net (J.H.S.); shpark2@morechem.net (S.H.P.); dhwang@morechem.net (D.H.W.); hkkwon@morechem.net (H.K.K.); 2 Morechem. Co., Ltd., Gangnam-daero, Seocho-gu, Seoul 06628, Republic of Korea

**Keywords:** Meloside A, dermal papilla, anti-hair loss, androgen receptor, androgenic alopecia

## Abstract

Androgenetic alopecia (AGA) is associated with dihydrotestosterone (DHT)-induced apoptosis in human dermal papilla cells (HDPCs) via androgen receptor (AR) upregulation. This study aimed to evaluate the potential of *Cucumis melo* var. *makuwa* leaf extract (CLE) to attenuate these DHT-mediated effects in HDPCs. HDPCs were treated with CLE, and DHT-induced apoptosis and AR expression were assessed. High-performance liquid chromatography coupled with electrospray ionization tandem mass spectrometry (HPLC–ESI–MS) identified Meloside A as the principal bioactive constituent within CLE. CLE significantly attenuated DHT-induced apoptosis in HDPCs, demonstrating a 57.74% reduction at 1000 ppm. Mechanistically, Meloside A inhibited DHT-stimulated AR nuclear translocation and reduced AR protein expression. Furthermore, Meloside A decreased the expression of downstream target genes at 100 ppm, showing a 16.27% reduction in IL-6, a 26.55% reduction in TGF-β1, and a 35.38% reduction in DKK-1. Additionally, Meloside A significantly inhibited ROS generation within DHT-stimulated HDPCs by 45.45% at 100 ppm. These findings suggest that Meloside A, isolated from CLE, exerts anti-AGA effects by modulating AR nuclear translocation and gene expression. This highlights its potential as a therapeutic agent for AGA and provides a basis for developing novel therapeutic strategies for hair loss.

## 1. Introduction

Hair loss represents a significant global health burden [1], with a multifactorial etiology encompassing various factors, including aging, nutritional deficiencies, hormonal imbalances, disease states, and stress [2,3,4,5,6]. Androgenetic alopecia (AGA) constitutes the most prevalent form of hair loss, affecting individuals of both sexes [7]. The dihydrotestosterone (DHT) hormone, derived from the 5α-reductase-catalyzed conversion of testosterone, plays a pivotal role in this pathogenic process. DHT interacts with the androgen receptor (AR) in hair follicles, shortening the hair growth cycle and promoting follicular miniaturization [8,9].

Upon DHT binding, AR functions as a transcription factor, enhancing the expression of target genes and modulating cell cycle progression within hair follicles, ultimately leading to follicular degeneration. Among these target genes are Interleukin-6 (IL-6), Dickkopf WNT Signaling Pathway Inhibitor 1 (DKK-1), and Transforming Growth Factor-beta1 (TGF-β1) [10,11,12,13], whose increased expression contributes to AGA. This increased AR expression sensitizes cells to DHT, potentially worsening hair loss. Human dermal papilla cells (HDPCs) from androgen-sensitive scalp regions (e.g., beard, axilla, pubic region, and vertex) display higher AR levels compared to those from androgen-insensitive non-balding occipital scalp follicles [9,14,15]. Therefore, targeting AR activity represents a promising therapeutic avenue for AGA.

Conventional pharmacological therapy for AGA, such as the FDA-approved agents minoxidil and finasteride, necessitate prolonged administration and are frequently associated with undesirable adverse events. Consequently, there is a growing impetus to investigate alternative therapeutic strategies, particularly emphasizing the favorable safety profiles of naturally derived compounds as potential replacements for established pharmacotherapies [16]. In line with this, recent studies have demonstrated that the natural compound cyanidin 3-O-arabinoside inhibits DHT-induced AR nuclear translocation and cp-asiAR effectively blocks androgen-mediated biological responses [17,18]. These findings underscore the therapeutic potential of targeting AR genes, which represent crucial mediators induced by androgens.

The oriental melon (*Cucumis melo* var. *makuwa*), known as “chamoe” in Korea, is a widely cultivated fruit in Asia. Traditionally used in Korean folk medicine for various ailments, it is reported to exert anti-microbial, anti-oxidative, and chemopreventive effects [19,20,21]. These activities stem from its rich phytochemical composition, including flavone glycosides and terpenes with potential pharmacological functions [20].

While previous research has demonstrated the effects of hot water extracts from *C. melo* var. *makuwa* on human hair growth ex vivo using hair follicle organ culture [21], the precise mechanisms of action, especially those related to androgen receptor signaling, are currently unknown.

In this study, we investigated the modulatory effects of *Cucumis melo* var. *makuwa* leaf extract (CLE) on DHT-stimulated AR expression and delineated the inhibitory mechanism of Meloside A, a constituent of CLE, on AR nuclear translocation activity in HDPCs, with the ultimate objective of elucidating its potential cosmetic and therapeutic application in the context of AGA.

## 2. Materials and Methods

### 2.1. CLE Preparation

Dried leaves of *C. melo* var. *makuwa*, purchased from Mins Market (Seoul, Republic of Korea), were subjected to hot water extraction. Briefly, 100 g of dried leaves was extracted with 1500 mL of water at 70 °C for 4 h. Following extraction, the extract was concentrated using a rotary evaporator (Eyela, Tokyo, Japan). The resulting dried extract was then weighed to determine the extraction yield, and this dried extract was used for subsequent experiments.

### 2.2. Reagents and Plasmids

Meloside A was purchased from Chemface (Wuhan, China). Dihydrotestosterone and minoxidil were purchased from Sigma Aldrich (St. Louis, MO, USA). CPPI (3-(4-chlorophenyl)-6,7-dihydro-5H-pyrrolo [1,2-a] imidazole) was purchased from ChemSpace (Monmouth Junction, NJ, USA). The pEGFP-C1-AR expression plasmid (Addgene plasmid #28235) was obtained from Addgene (Watertown, MA, USA).

### 2.3. Culture of Human Dermal Papilla Cells (HDPCs)

HDPCs, purchased from Cell Engineering For Origin (Seoul, Republic of Korea), were cultured at 37 °C in a 5% CO_2_ incubator and maintained in Dulbecco’s Modified Eagle Medium (DMEM) supplemented with 10% fetal bovine serum (FBS) and 1% penicillin/streptomycin (Welgene, Gyeongsan, Republic of Korea).

### 2.4. pEGFP-C1-AR Subcellular Localization

HDPCs were transfected with a pEGFP-C1-AR expression vector. After transfection, cells were fixed and permeabilized with ice-cold 4% paraformaldehyde for 15 min at room temperature. Fluorescence microscopy was performed using an EVOS^®^ FL auto microscope (20×, 40× objective, P4734; Thermo Fisher Scientific, Waltham, MA, USA), and images were processed using EVOS^®^ software (version 1.4). The subcellular localization of GFP-AR (nuclear vs. cytosolic) was determined by manual counting of 200 cells per condition. The proportion of cells exhibiting nuclear localization was then calculated.

### 2.5. Enzyme-Linked Immunosorbent Assay (ELISA)

Culture supernatants were collected, and protein levels of DKK-1, TGF-β1, and IL-6 were determined using human DKK-1 (DY1906), TGF-β1 (DY240-05), and IL-6 (DY206-05) Duo Set ELISA Kits (R&D Systems, Minneapolis, MN, USA) according to the manufacturer’s instructions. Briefly, 96-well plates were coated with capture antibodies against human DKK-1, TGF-β1, and IL-6 and incubated overnight at room temperature. After blocking with 1% blocking buffer (BSA) in PBS (pH 7.2), samples were added in triplicate and incubated for 2 h at room temperature. Following incubation, detection antibodies specific for human DKK-1, TGF-β1, and IL-6 were added. Washing steps were performed after each incubation. Subsequently, streptavidin-horseradish peroxidase (HRP) was added, followed by further washing and incubation. Finally, substrate solution was added, and absorbance was measured at 450 nm using an EON microplate reader (BioTek, Winooski, VT, USA). The experiment was performed with n = 3 participants.

### 2.6. Western Blot Analysis

Cell lysates were prepared in 2× Laemmli sample buffer (Bio-Rad, Hercules, CA, USA), and total protein concentration was determined using the Bradford assay (Bio-Rad, Hercules, CA, USA), following the manufacturer’s protocol. Proteins were separated by sodium dodecyl sulfate-polyacrylamide gel electrophoresis and transferred to poly-vinylidene fluoride membranes. Membranes were blocked with 4% skim milk in tris-buffered saline supplemented with Tween-20 and then incubated with primary antibodies against AR (ab133273; Abcam, Cambridgeshire, CB, UK; 1:1000) and actin (NB600-501; NOVUS Biologicals, Littleton, CO, USA; 1:10,000). After washing, membranes were incubated with HRP-conjugated secondary antibodies (Cell signaling Technology, Danvers, MA, USA). The experiment was performed with n = 3 participants.

### 2.7. HPLC–ESI–MS Analysis

The standard solution of Meloside A was prepared in methanol at a concentration of 100 μg/mL. The extract of *Cucumis melo* leaves was also dissolved in methanol to a concentration of 1000 μg/mL prior to analysis. LC/MS analyses were conducted using a Waters separation module coupled to a Waters SQD mass spectrometer (Waters, Milford, MA, USA). The system included a binary pump, autosampler, degasser, and photodiode array (PDA) detector. Chromatographic separation was performed on a Thermo Scientific Acclaim 120 C18 column (2.1 × 150 mm, 3 μm) at a column temperature of 25 °C. Gradient elution was employed using a mobile phase consisting of water with 0.1% formic acid (solvent A) and methanol with 0.1% formic acid (solvent B). The gradient program was as follows: 80% A/20% B (0 min), 60% A/40% B (30 min), 100% B (35 min), and 80% A/20% B (36 min). The flow rate and injection volume were 0.4 mL/min and 3 μL, respectively. The column effluent was introduced into the mass spectrometer source via electrospray ionization (ESI) operating in positive (+) and negative (−) ion modes, with an *m*/*z* scan range of 200–800. Argon was utilized as a nebulizer and curtain gas. Mass spectrometer source parameters were as follows: capillary voltage, 3.5 kV; cone voltage, 30 V; extractor cone voltage, 3 V; RF lens voltage, 0.1 V; source temperature, 120 °C; desolvation temperature, 300 °C; desolvation gas flow rate, 600 L/h; and cone gas flow rate, 50 L/h. Mass spectra were acquired and analyzed using Mass Lynx software version 4.1 (Waters, Milford, MA, USA).

### 2.8. Statistical Analysis

Data were presented as mean ± standard error of the mean (SEM) from at least three independent experiments. Statistical analysis was performed using Brown–Forsythe and Welch’s one-way ANOVA tests, followed by Dunnett’s T3 post hoc comparisons. Differences were considered statistically significant at *p* < 0.05.

## 3. Results

### 3.1. Evaluation of the Proliferative Capacity in Human Follicle Dermal Papilla Cells (HDPCs)

**Figure 1:**
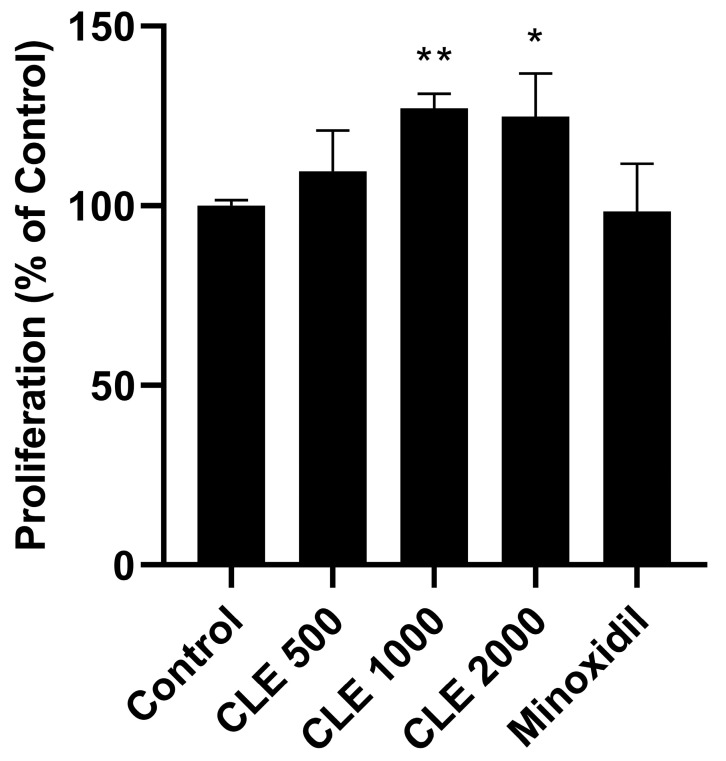
HDPCs proliferation was assessed after 48 h of treatment with various concentrations of CLE (500, 1000, and 2000 μg/mL) or minoxidil (positive control) using the CCK-8 assay. Data are shown as mean ± SEM from at least three independent experiments. * *p* < 0.05, ** *p* < 0.01. CLE, *Cucumis melo* var. *makuwa* leaf extract; CCK-8, cell counting kit-8.

While previous studies have shown that CLE promotes hair growth in vivo using a C57BL/6 mice model [21], the molecular mechanisms by which CLE delays catagen progression remain largely unknown. To address this, we first investigated the effect of CLE on HDPCs proliferation using CCK-8 (Cell Counting Kit-8) in serum-free conditions, comparing it to minoxidil as a positive control. As shown in Figure 1, CLE significantly enhanced cell proliferation at a concentration of 1000 μg/mL. This concentration was used for all subsequent experiments.

### 3.2. CLE Inhibits DHT-Induced AR Expression

**Figure 2:**
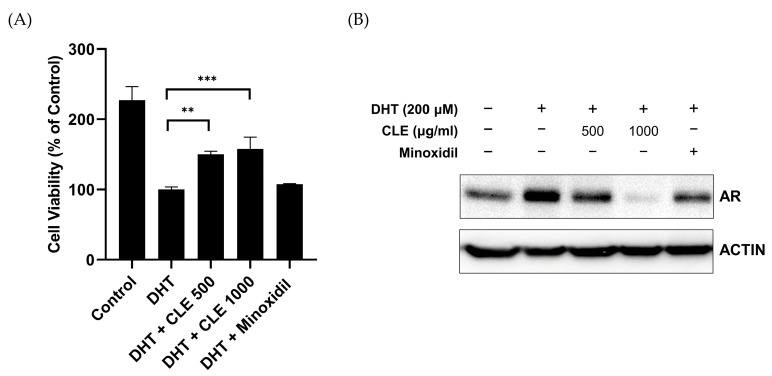
(**A**,**B**) HDPCs were co-treated with CLE or minoxidil in the presence of DHT for 24 h. (**A**) Cell viability was determined using the CCK-8 assay. (**B**) AR protein expression was analyzed by western blotting. Data are shown as mean ± SEM from at least three independent experiments. ** *p* < 0.01, *** *p* < 0.001. DHT, dihydrotestosterone; CLE, Cucumis melo var. makuwa leaf extract; CCK-8, cell counting kit-8.

To determine whether CLE influences DHT-induced cell death and AR expression, a critical factor in male pattern baldness, DHT-treated HDPCs were incubated with CLE or the known AR inhibitor minoxidil [22]. CLE treatment effectively reduced DHT-induced cell death in a concentration-dependent manner within 500 and 1000 μg/mL (Figure 2A). We further assessed the impact of CLE on the expression of proteins relevant to AGA following DHT stimulation. CLE reduced AR expression to levels similar to those observed in minoxidil-treated cells (Figure 2B). These findings suggest that CLE promotes HDPC proliferation and modulates DHT-induced AR expression, thereby inhibiting cell death.

### 3.3. Meloside A Identification in CLE

To identify potential anti-androgenic regulators within CLE, we performed HPLC–ESI–MS analysis, as detailed in the Materials and Methods section. LC–MS data, including retention times, PDA chromatograms, *m*/*z* values for [M + H]^+^, [M + H]^−^, and fragmentation patterns from the base peak chromatogram, were manually curated. Previous studies have reported the isolation of Meloside A and its caffeoyl esters from melon leaves [20], suggesting that these compounds may be present in CLE.

**Figure 3:**
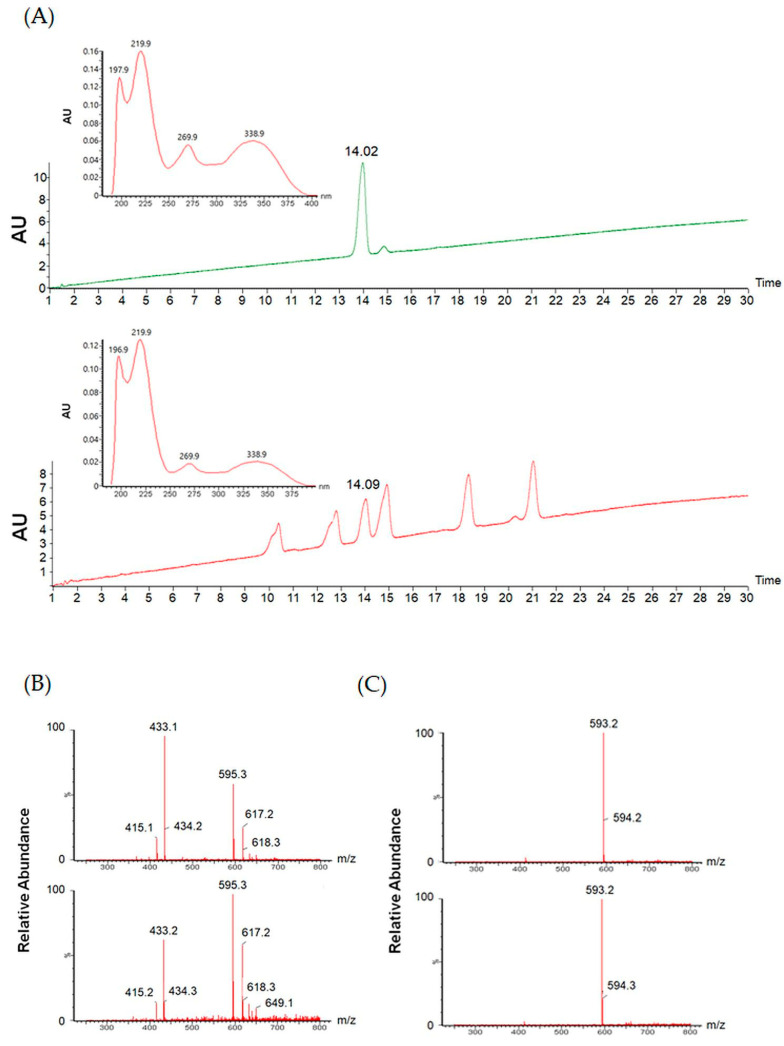
(**A**) Comparison of LC chromatograms obtained using a photodiode array detector for Meloside A (top) and CLE (bottom). (**B**) Comparison of ESI mass spectra (positive ion mode) for Meloside A (top) and CLE (bottom). (**C**) Comparison of ESI mass spectra (negative ion mode) for Meloside A (top) and CLE (bottom). CLE, *Cucumis melo* var. *makuwa* leaf extract.

The UV spectrum of CLE showed characteristic absorption peaks at 270 nm and 315 nm, indicative of phenylpropanoids [23]. As Meloside A exhibited similar UV spectral properties, and considering preliminary ESI data, we prioritized its analysis. To confirm the presence of Meloside A in CLE, HPLC–ESI–MS analysis was performed Comparison of retention times on the resulting chromatograms identified a corresponding peak in CLE. This peak was further analyzed by UV spectroscopy, and its identity was confirmed by comparison with the Meloside A standard (Figure 3A). The identity of Meloside A in CLE was further corroborated by comparison of the LC–MS fragmentation pattern in both positive and negative ESI modes (Figure 3B,C).

Spectroscopic (UV) and mass spectrometric (ESI) data were used to identify and con-firm the presence of Meloside A in CLE. Subsequent quantitative analysis determined the concentration of Meloside A to be 80 ± 20 mg/g, with the high standard deviation attributed to biological variation depending on the harvest time of the leaves.

### 3.4. Meloside A Inhibits DHT-Induced AR Nuclear Translocation

**Figure 4:**
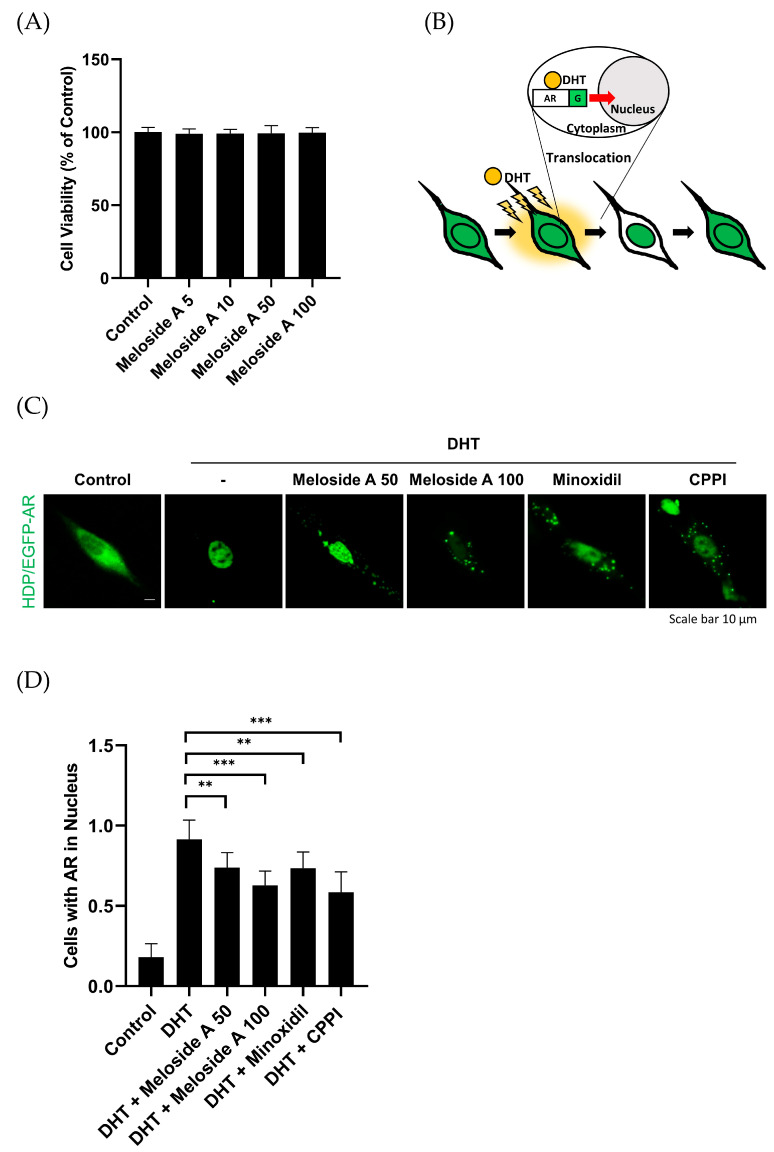
(**A**) Cytotoxic effects of Meloside A on HDPCs. Cell viability was assessed after 24 h of treatment with various concentrations of Meloside A (5, 10, 50, and 100 μg/mL) using the CCK-8 assay. (**B**) Schematic representation of the pEGFP-AR system used to monitor AR translocation. (**C**,**D**) Effect of Meloside A on DHT-induced AR nuclear translocation. EGFP-AR-expressing HDPCs were treated with Meloside A (50 or 100 μg/mL), minoxidil (100 μM), or CPPI (30 μM) for 6 h following DHT stimulation. (**C**) Representative fluorescence microscopy images showing the subcellular localization of pEGFP-AR. Scale bar, 10 μm. (**D**) Quantification of cells exhibiting nuclear AR localization. Data are presented as mean ± SEM from at least three independent experiments. ** *p* < 0.01, *** *p* < 0.001. DHT, dihydrotestosterone; CPPI, 3-(4-chlorophenyl)-6,7-dihydro-5H-pyrrolo [1,2-a] imidazole.

Previous studies have established that androgen binding to AR triggers AR nuclear translocation, resulting in increased AR expression and subsequent transcriptional regulation of androgen-responsive genes, including DKK-1 [18,24]. Here, we investigated the effect of Meloside A, an active CLE compound, on DHT-induced AR nuclear translocation. To determine a non-cytotoxic concentration of Meloside A, we assessed HDPCs viability. Meloside A at 100 μg/mL did not induce significant cytotoxicity (Figure 4A). DHT-stimulated EGFP-AR-expressing HDPCs were then treated with Meloside A or CPPI, a known inhibitor of AR nuclear translocation [25]. Meloside A inhibited DHT-induced AR nuclear translocation in a dose-dependent manner, exhibiting similar efficacy to CPPI (Figure 4C,D). These results suggest that Meloside A modulates AR nuclear translocation in DHT-stimulated HDPCs.

### 3.5. Meloside A Modulates AR Downstream Signaling and Protects Against Oxidative Stress

**Figure 5:**
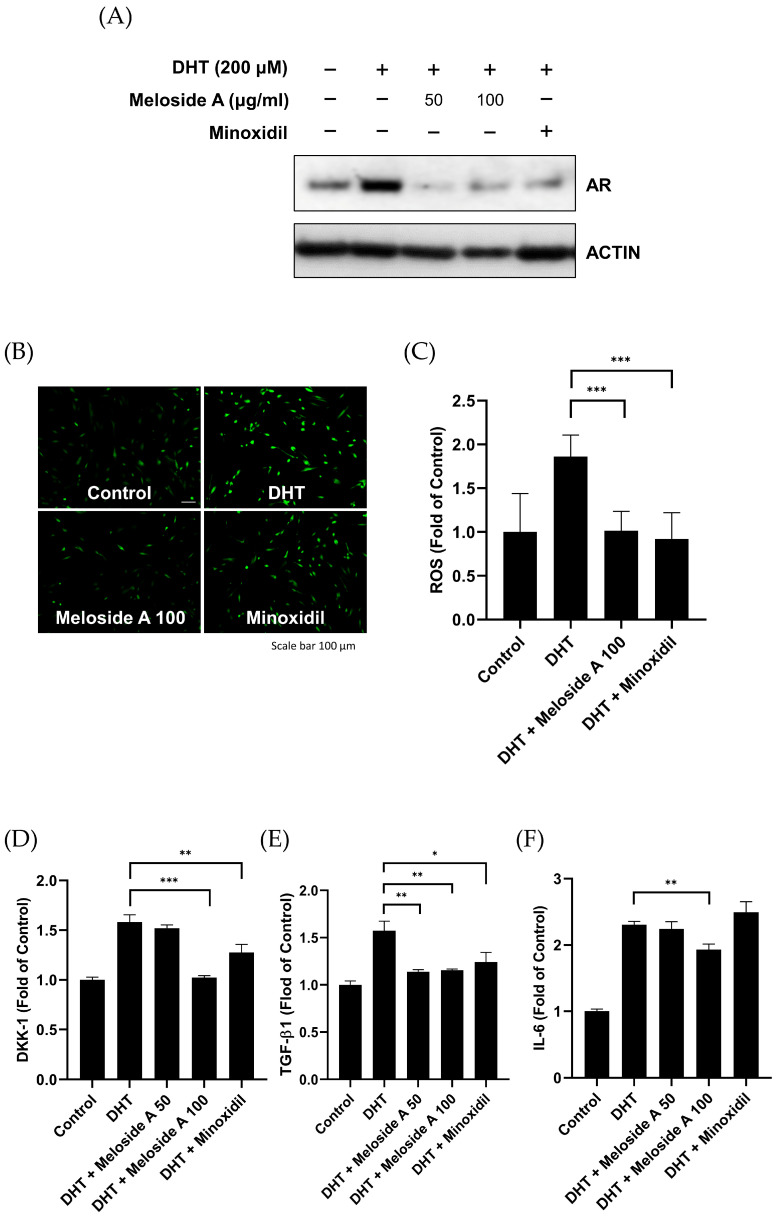
(**A**–**F**) HDPCs were treated with Meloside A (50 or 100 μg/mL) and minoxidil (100 µM) in the presence of DHT (200 µM) for 24 h. (**A**) AR protein levels were determined by western blotting. (**B**,**C**) Protective effect of Meloside A on DHT-induced ROS production. Cells were stained with DCF-DA for 30 min, and representative fluorescence microscopy images were obtained. Scale bar, 10 µm. ROS levels were quantified using ImageJ (version 1.54k). (**D**–**F**) Levels of DKK-1 (**D**), IL-6 (**E**), and TGF-β1 (**F**) in the culture medium were measured by ELISA after 24 h of co-treatment. Data are presented as mean ± SEM from at least three independent experiments. * *p* < 0.05, ** *p* < 0.01, *** *p* < 0.001. DHT, dihydrotestosterone; ROS, reactive oxygen species.

It is established that HDPCs respond to androgens by producing various factors, contributing to hair loss [24]. We therefore investigated the impact of Meloside A on AR downstream signaling in DHT-stimulated HDPCs. Initially, we assessed the effect of Meloside A on AR protein expression. Treatment with Meloside A at concentrations of 50 and 100 μg/mL significantly reduced DHT-induced AR protein expression (Figure 5A). Given that DHT induces apoptosis through the accumulation of reactive oxygen species (ROS) [26], we evaluated the protective effect of Meloside A against DHT-induced oxidative stress by co-incubating cells with Meloside A and 200 μM DHT for 24 h. Meloside A significantly attenuated ROS levels, similar to the effect of minoxidil (Figure 5B,C). Furthermore, we examined the influence of Meloside A on the secretion of AGA-related proteins DKK-1, IL-6, and TGF-β1 using ELISA. Co-incubation with Meloside A for 48 h resulted in a significant decrease in the secretion of DKK-1, IL-6, and TGF-β1 compared to DHT treatment alone (Figure 5D–F). These results suggest that Meloside A modulates AR downstream signaling pathways and protects against DHT-induced oxidative stress in HDPCs.

## 4. Discussions

*Cucumis melo* var. *makuwa* extract has a history of use in traditional Korean herbal medicine due to its antioxidant properties [19,20]. Furthermore, the skin and seeds of this melon have been shown to possess diverse biological activities, including anti-inflammatory, anticancer, antibacterial, hepatoprotective, and immunomodulatory effects, suggesting potential therapeutic applications in cardiovascular diseases, diabetes, and edema [27]. While recent studies have shown that CLE promotes hair growth by shortening the telogen phase [21], the specific active components and their mechanisms of action in inhibiting hair loss remain to be elucidated.

Recently, other groups showed that modulating AR expression has emerged as a promising therapeutic strategy for AGA, with localized AR downregulation demonstrating a favorable safety profile [28,29]. Treatment of vitisin A inhibits AR expression and apoptosis by DHT. In addition, application of vitisin A cream effectively alleviated AGA in mice [30]. Moreover, injection of AR-targeting asiRNA promoted hair growth in mouse models with DHT-induced AGA [18]. We therefore investigated the interaction between CLE and AR. Treatment with CLE effectively inhibited AR expression in DHT-induced HDPCs (Figure 2).

To identify potential anti-androgen receptor molecules within CLE, we performed comparative analyses of UV and MS–ESI data. Analysis of CLE identified several phytochemical peaks. Based on preliminary screening results and a literature review, Meloside A was selected as a candidate compound for further investigation into its anti-androgenic properties.

Meloside A is a phenylpropanoid that is found in several plant species, including melon, jujube, barley, and passion flower. Meloside A has demonstrated protective effects against UV-B radiation and pathogens [31,32,33]. Phenylpropanoids exhibit anti-hair loss effects. Various phenylpropanoids, including rosmarinic acid and quercetin, attenuate cell death caused by testosterone [34]. While other phenylpropanoids like vitisin A have shown potential anti-androgenic properties [30], the specific effect of Meloside A on AR regulation has not been previously investigated. In this study, we investigated whether Meloside A treatment would inhibit AR-mediated effects in HDPCs.

Interestingly, it was recently reported that as a ligand-dependent transcription factor, AR regulates the expression of androgen-responsive genes, with nuclear translocation being a critical regulatory step [18]. Inhibiting DHT-induced AR nuclear translocation offers a potential avenue for hair loss treatment. While the precise molecular pathogenesis of AGA is complex, key androgen-inducible mediators such as TGF-β1, IL-6, and DKK-1 are known to play significant roles. These molecules contribute to follicular regression by promoting catagen induction (TGF-β1) and inhibiting hair elongation (IL-6 and DKK-1), and their expression levels are widely utilized as surrogate markers for evaluating AGA treatment efficacy [10,11]. Given the limited exploration of targeting AR nuclear localization, we developed a GFP-AR-based monitoring system. We demonstrated that Meloside A effectively inhibits AR nuclear translocation in HDPCs. Consistent with this inhibitory effect on AR activity and nuclear translocation, Meloside A treatment subsequently led to a significant reduction in the expression of these critical downstream proteins, such as IL-6, TGF-β1, and DKK-1 (Figure 4 and Figure 5). This finding suggests that Meloside A may counteract AGA pathogenesis by modulating the expression of these key mediators.

Enzalutamide, a known anti-androgen, competitively binds to AR [35]. We hypothesized that Meloside A may similarly compete with DHT for AR binding, thereby inhibiting nuclear translocation. Alternatively, Meloside A may target AR-associated cofactors involved in intracellular localization. Given that heat shock protein 90 (HSP90) defect has been observed in AGA patients and that HSP90 knockdown inhibits HDPC growth [36], and considering that HSP90 dissociation from AR promotes nuclear translocation [37], we propose that Meloside A may also modulate HSP90 activity. Further studies are needed to elucidate the precise mechanisms by which Meloside A affects AR binding and HSP90 activity.

However, the findings of this study, primarily based on simplified in vitro cell models, should be interpreted in light of several limitations. These models inherently lack the complex physiological microenvironment of the hair follicle in vivo, including essential components such as vascular supply and interactions with diverse cell types. While previous research indicates that AR depletion reduces the proportion of telogen hair in ex vivo human hair follicles [19], our in vitro based study did not directly assess whether Meloside A exhibits a similar telogen-reducing effect in this specific context. Furthermore, although we propose potential mechanisms, the precise molecular interactions of Meloside A with AR and related proteins require further detailed investigation. Therefore, validation of our findings and evaluation of efficacy in more physiologically relevant models of AGA, such as ex vivo human hair follicle cultures or in vivo animal models, are necessary to confirm the therapeutic potential of Meloside A for hair loss treatment.

**Figure 6:**
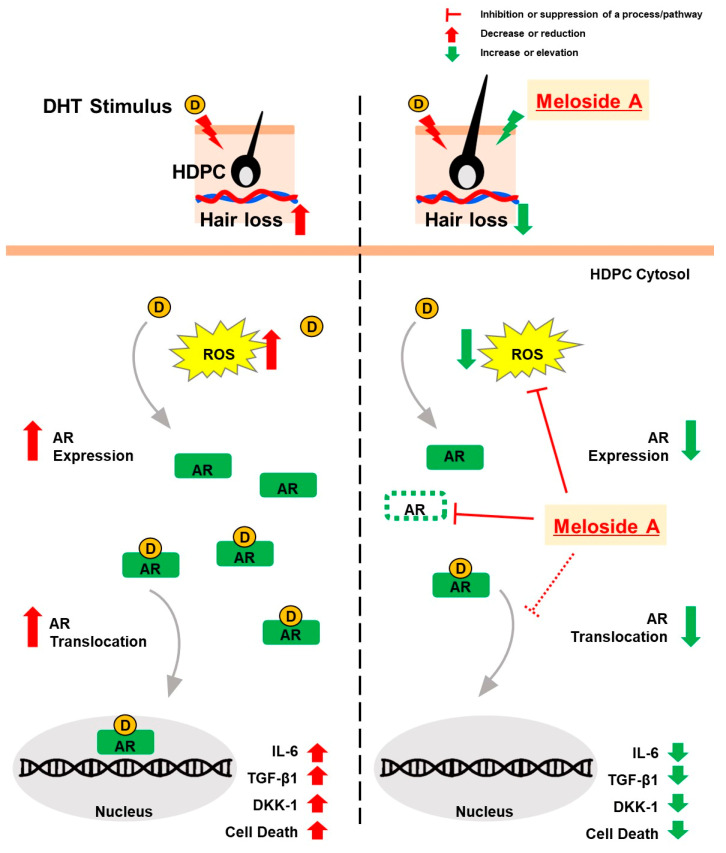
Meloside A treatment in DHT-stimulated HDPCs demonstrates in vitro effects by targeting the AR signaling pathway, leading to suppressed AR trans-activation and expression, reduced ROS production, and inhibited expression of AR downstream targets (DKK-1, TGF-β1, IL-6).

In conclusion, Meloside A treatment in DHT-stimulated cells suppressed AR transactivation, reduced AR expression, and attenuated ROS production. Furthermore, Meloside A inhibited the expression of AR downstream targets, including DKK-1, TGF-β1, and IL-6 (Figure 6). While further research is needed to fully elucidate its mechanism of action and potential side effects, these findings suggest that Meloside A exhibits effects similar to those of minoxidil in vitro. These data suggest that Meloside A may have potential for cosmetic applications and warrants further investigation as a therapeutic candidate for hair loss.

## Data Availability

The raw data supporting the conclusions of this article will be made available by the corresponding author upon request.
